# Corroborating evidence refutes batch effect as explanation for fetal bacteria

**DOI:** 10.1186/s40168-020-00948-0

**Published:** 2021-01-12

**Authors:** E. Rackaityte, J. Halkias, E. M. Fukui, V. F. Mendoza, C. Hayzelden, E. D. Crawford, K. E. Fujimura, T. D. Burt, S. V. Lynch

**Affiliations:** 1 Division of Gastroenterology, Department of Medicine, University of California, San Francisco, San Francisco, CA USA; 2 Biomedical Sciences Graduate Program, University of California, San Francisco, San Francisco, CA USA; 3 Division of Neonatology, Department of Pediatrics, University of California, San Francisco, San Francisco, CA USA; 4 Eli and Edythe Broad Center of Regeneration Medicine and Stem Cell Research, University of California, San Francisco, San Francisco, CA USA; 5 College of Science and Engineering, San Francisco State University, San Francisco, CA USA; 6 Chan Zuckerberg Biohub, San Francisco, CA USA; 7 Department of Microbiology and Immunology, University of California, San Francisco, San Francisco, CA USA; 8 Genentech, South San Francisco, CA USA; 9 Duke University School of Medicine, Durham, NC USA

While next-generation sequencing has spurred radical growth in the microbiome field, its limitations have been exposed in the interrogation of low-burden microbiomes [1, 2]. Thus, studies of such niches require exceptional rigor in sample handling, data generation, analyses and interpretation, and must provide multiple independent corroborating lines of evidence to reject or support proposed hypotheses. De Goffau et al report a batch effect in the 16S rRNA dataset included as part of our recently published study [3] and call into question the validity of the presence of *Micrococcus* in a subset of human fetal intestinal samples. Unlike other studies in the field, the 16S rRNA data in our study was not used as the sole evidence for bacterial presence in utero, but rather as a means to classify bacteria that accounted for the sparse signal initially observed in fetal intestinal specimens by corroborating qPCR and Fluorescent in situ Hybridization (FISH) analyses, and to guide culture, isolation and characterization of these microbes. Beyond 16S rRNA-based bacterial classification, our study included multiple lines of direct and indirect evidence for a highly limited bacterial signal in subsets of human fetal meconium, including scanning electron microscopy as well as differentiating intestinal mucosal immune responses, including transcripts and proteins induced by bacteria.

**Fig. 1:**
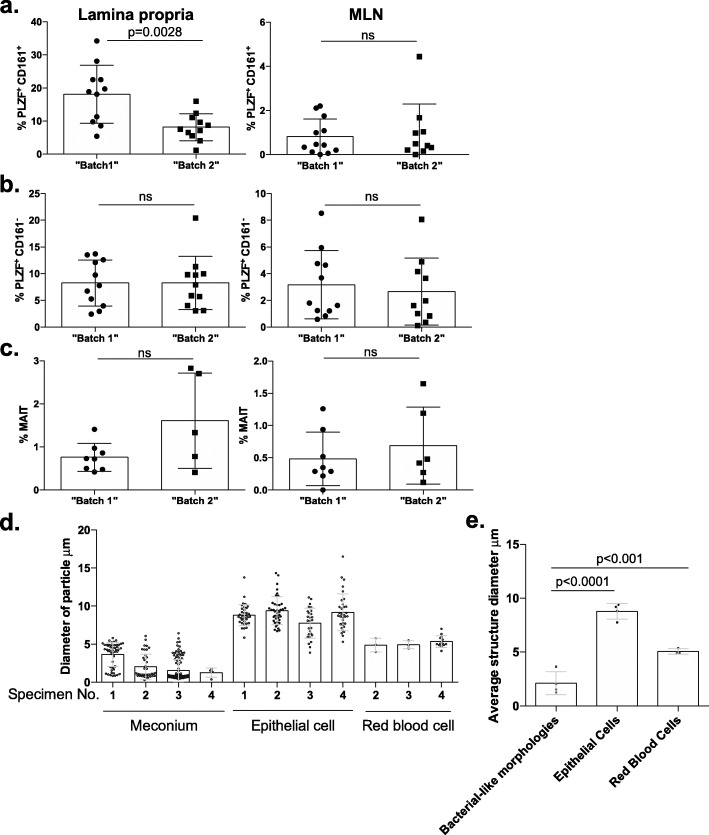
Fetal intestinal cell measures refute the presence of technical batch effects. Proportion of **a** PLZF^+^ CD161^+^, **b** PLZF^+^ CD161^−^ T cells among live, TCRβ^+^, Vα7.2^−^, CD4^+^ cells and **c** mucosa-associated invariant T cells (MAITs; CD161^+^ among live, TCRβ^+^, Vα7.2^+^ cells) in intestinal lamina propria (left) or mesenteric lymph node (MLN, right). t-test for significance, where each dot is a biologically independent fetal sample. **d** Diameter (μm) and **e** average structure diameter (μm) of bacterial-like morphologies in meconium, epithelial cells, and red blood cells as detected by scanning electron microscopy across up to four biologically independent fetal samples. Each dot represents a cell structure in (**d**) and a fetal sample in (**e**) One-way ANOVA with Tukey’s test for multiple comparisons was used to calculate significance in (**e**)

De Goffau and colleagues [4] performed a reanalysis of non-normalized raw 16S rRNA data provided in the supplemental methods of our manuscript. The authors included all samples with ≥100 sequence reads in their re-analysis and report a batch effect based on this dataset, such as presented in their Figure 1c. Inclusion of samples with lower sequence reads in a study of very low bacterial burden can artificially inflate false negatives due to inadequate community coverage – this is particularly pertinent since “Batch 2” samples had significantly lower sequence reads per sample than those of “Batch 1” (excluding mock controls; median read depth “Batch 2” = 19,601, “Batch 1*”* = 39,194; *P* < 0.0001; specimen read depth available in Supplemental Table 2 of our original manuscript), plausibly explaining the higher rate of *Micrococcaceae*-negative samples in “Batch 2”. Because our qPCR- and FISH-based analyses of fetal specimens had indicated a sparse, low-burden bacterial presence, the 16S rRNA analysis performed and reported in our study used a multiply rarefied dataset (to ensure that the 16S rRNA profiles were representative) and included only those samples with ≥1000 16S rRNA sequence reads to permit confident detection of bacterial signals. Normalization of read-depth is recommended for analysis of zero-inflated microbiome data and enables clustering of samples according to biological metadata [5], yet this was not performed by De Goffau and colleagues. Moreover, the batch effect described by De Goffau which the authors claim explain the fetal *Micrococcus* 16S rRNA signal in Figure 1 and Figure 2, is predicated upon Principal Component Analysis (PCA), an ordination method based on Euclidian distance which assumes linear relationships and a normal data distribution. These assumptions are violated in most biological datasets, but particularly in zero-inflated, low-burden 16S rRNA data [6–9] such as that produced in the study of human in utero samples. As a result, multiple studies have indicated that application of PCA to such datasets results in “*false distributions and outputs*” and is considered inappropriate [10]. Application of PCA primarily accounts for the observation that almost all variation in the non-normalized 16S rRNA dataset can be explained on PC1 and 2, an uncommon finding when appropriate distance matrices and ordination approaches are applied that consider the nature and distribution of the data.

Contrary to De Goffau and colleagues’ assumption, and as stated in our methods section, samples were not processed in batches. Indeed, as stated in our methods [3] many measures were put in place to control for batch effects, including a single operator collecting all samples, use of a single molecular grade buffer batch for all extractions (no commercial kits were used), inclusion of a mock community on each amplification PCR plate, and generation of 16S rRNA sequence data on a single sequencing run. In addition to technical controls, supplementary biological and technical controls were added into the sample collection protocol following the publication of a manuscript by Lauder et al in 2016 [11], indicating the need for such samples. This did not change the handling of specimens during collection, but rather allowed for additional controls to be collected at the time of specimen acquisition. We acknowledge that these controls were not collected in parallel with the initial specimens but also point to Extended Data 3i of our manuscript [3] in which we demonstrate that the large majority of meconium 16S rRNA profiles (with the exception of 2 specimens) cluster distinctly from controls (*n* = 48) using appropriate ordination methods. We also note that while *Micrococcus* was indeed identified as enriched in earlier specimens in our study, specimens utilized for scanning electron microscopy and fluorescent in situ hybridization studies, which independently evidence microbial signal and cells respectively, were collected after those used for 16S rRNA analyses. Moreover, 16S rRNA reads for *Micrococcaceae* OTU10, found to be enriched in meconium specimens compared to a multitude of technical (*n* = 48) and biological (*n* = 35) control samples processed in parallel, were not exclusively detected in “Batch 1”, but existed in both “Batches” (Extended Data Figure 3h of our original manuscript). This was also true of 16S rRNA sequences of *Lactobacillus*, which was also significantly enriched in meconium specimens and detected in both “Batches”. In addition, 16S rRNA sequences with 100% sequence identity to the *Micrococcus luteus* isolated from fetal meconium specimens exist in *both* “Batch 1 and 2”, further refuting the claims made by De Goffau and colleagues.

To claim that Batch 1 and 2 are due to technical variance (indicative of batching that occurred during sample processing), one must not find other biological metadata that could explain the batch effect. We note that at the time of collection, specimens were separated into distinct aliquots for 16S rRNA analyses, RNASeq, flow cytometry and bacterial isolation, thus the latter three aliquots never came into contact with reagents used in 16S rRNA analyses. We reanalyzed our metadata, binning samples based on the De Goffau batch classification and find that “Batch 1”, which is enriched for *Micrococcaceae*, exhibits a significantly greater proportion of PLZF^+^ CD161^+^ T cells in the lamina propria (Fig. 1a) and that differences between “Batch 1” and “Batch 2” were also detected in epithelial RNAseq signatures. These immune datasets were generated independently of the 16S rRNA data, were not influenced by spurious contamination and provide support for a distinct intestinal immune landscape in samples enriched for *Micrococcaceae* (enriched in “Batch 1”). No change in specimen collection or in the immune cell isolation protocol occurred, pointing to specific underlying biological differences that result in paired meconium, epithelial, and T cell profile changes.

To claim that Batch 1 and 2 are due to technical variance one must also find that additional biological data supports the observed batch effect (which would be indicative of specimen collection issues upstream of the data generation process). Our study provides evidence for significant differences in the frequency of mucosal PLZF^+^ CD161^+^ T cells in samples enriched for *Micrococcaceae* which predominate “Batch 1”. To assess whether other immune data was also influenced by the proposed batching, we examined additional mucosal T cell subsets and those at distinct sites (e.g. mesenteric lymph node) measured contemporaneously. Neither mucosal PLZF^+^ T cells lacking CD161 expression (Fig. 1b) nor mucosal-associated invariant T cells (MAIT; defined as live, TCRβ^+^, Vα7.2^+^, CD161^+^; Fig. 1c) exhibited significant differences across “Batch 1 and 2” in the lamina propria. Moreover, all three of these distinct T cell populations did not significantly differ across “*Batch 1* and *2*” in the mesenteric lymph node (Fig. 1a-c). Thus, for De Goffau and colleagues’ claims to be accurate, a confounder that exclusively influenced mucosal (but not lymphoid) PLZF^+^ CD161^+^ T cells must have been introduced. Given that PLZF^+^ CD161^+^ T cells are the exact T cell population modulated in vitro by the fetal (but not reference) *Micrococcus* isolate*,* and that this interaction both induces expression of the ligand for CD161 and inhibits their inflammatory function, we feel that these corroborating data serve to further refute the claim that *Micrococcus* presence in subsets of fetal meconium is due to a batch effect in the 16S rRNA dataset.

Our study included light microscopy and detected a eubacterial signal through fluorescent in situ hybridization. However, this approach requires thin-sectioning which dilutes a rare signal. Thus, we turned to scanning electron microscopy, which permits the ability to scan the surface of thick sections and obtain high magnification resolution of structures. We identified clusters of cellular structures consistent with the size of bacteria embedded in polysaccharide in intestinal meconium. We do not claim in our manuscript that these are *Micrococcus* specifically, but rather that they are consistent with the size and shape of bacterial cells. We have measured the size of these cellular structures localizing to meconium and find that they are 3.7 μm, 2.0 μm, 1.5 μm, 1.26 μm for specimens 1–4 respectively (Fig. 1d-e). We additionally measured the size of epithelial cells in our micrographs, which are identifiable by microvilli and clear cell boundaries and find that these are 8.8 μm, 9.4 μm, 7.75 μm, and 9.2 μm in diameter, respectively (Fig. 1d-e). The red blood cell is one of the smallest eukaryotic cells (second only to the sperm cell) and clearly identifiable by their round, enucleated shape. We measured red blood cells in three of our four specimens and found the average diameter to be 5.3 μm, 4.9 μm, and 4.9 μm, respectively. While the cellular structures in the panel upon which De Goffau and colleagues superimposed a micrograph of an environmental *M. luteus* may be larger, we observe coccoid structures well within the expected size for *M. luteus* (e.g. Specimen 3, Panel iii of our Fig. 1). Thus, our data support that these coccoid structures are within the range of bacterial cell proportions and not within the range of eukaryotic cells. We acknowledge that the identity of these cells is uncertain, and only claim in our manuscript that they exhibit a bacterial-like morphology. We also re-emphasize that samples utilized for light and electron microscopy were collected after analyses of those that underwent molecular analyses was completed, strongly refuting a temporal association with signal detection.

Finally, we note that fetal bacterial isolates were cultured from samples that never underwent DNA extraction and thus unexposed to potential contaminants associated with 16S rRNA analysis. We find sequences with 100% identity to our fetal *Micrococcus* isolate in *n* = 12 “Batch 1” and *n* = 8 “Batch 2” meconium samples, further refuting that *Micrococcaceae* are only present in “*Batch 1*.” We also note that the fetal *M. luteus* exhibits the ability to utilize placental hormones (which permit its limited growth) and persists within antigen presenting cells – features unique to this fetal strain not exhibited by phylogenetic relatives. De Goffau and colleagues cite studies using previously cultured *environmental M. luteus* (including ATCC ﻿4698, which we tested in our phenotyping experiments) to conclude that this species is easy to culture. However, reference strains simply refer to the first widely used isolate in the field and do not serve as accurate representations of the breadth of physiological diversity that can exist within a given species. First, the fetal strain Micro36 is not yellow but rather white, unlike the reference *M. luteus* strain, likely due to its loss of the carotenoid synthesis enzymes as indicated by whole genome sequencing and analysis. Second, we demonstrate, based on whole genome comparisons, that Micro36 clusters closer to other human isolates of *Micrococcus* than to environmental isolates of *Micrococcus* (our Extended Data Fig. 7). Third, we report that the human fetal *Micrococcus* strain exhibits vastly different physiological (including the ability to grow on pregnancy hormones and persist within antigen presenting cells) and immune modulation phenotypes that are not observed with the *environmental* reference. Likely contributing to these striking differences is the low level of genome wide similarity between *M. luteus* isolates of fetal and environmental origin (96.8%), which nears the new species boundary [12]. Finally, we demonstrated that the Micro36 16S rRNA V4 region falls within 97% similarity of the *representative* sequence within OTU10, which was binned as *Micrococcaceae* using a stringent bootstrap cut-off for taxonomic classification. The sequence reported in Extended Data Figure 5 is the *representative* sequence of OTU10 (as indicated in the figure legend) from sequences clustered at 97% identity using the USEARCH pipeline (as indicated in the methods). To investigate whether Micro36 was found among all sequences obtained from fetal meconium, we aligned all meconium 16S V4 sequences with Micro36 V4 sequence and found 100% sequence identity in 20 meconium specimens across both De Goffau “Batches”, including MM samples and the sample from which Micro36 was isolated, thus strongly refuting the author’s claim that isolated Micro36 was not sequenced by 16S V4 methods.

To fully address any hypothesis, one must generate orthogonal corroborating evidence which requires a holistic view of the data. De Goffau and colleagues reduce our body of data to two dimensions: “Batch 1” and “Batch 2” found in principal component 1 and 2 analysis of a non-normalized 16S rRNA dataset. We attempted to accept the sterile in utero hypothesis, but the multiple corroborating lines of evidence pointing to a limited bacterial presence prevented us from doing so. In our study we state, “*it is possible that the bacterial signal identified may arise from contamination from a source not investigated in this study, yet in our judgment, the corroborating evidence suggests that restriction of bacterial entry into the human fetal intestine is not absolute*” and we remain in favor of this interpretation of all of the data in hand.
